# Fungal diversity in larval diets of *Melipona interrupta*: Impacts on queen development and survival

**DOI:** 10.1590/1678-4685-GMB-2025-0021

**Published:** 2025-12-12

**Authors:** Luana Evillyn Vinente de Queiroz, Flavia da Silva Fernandes, Gabriel Garcês Santos, João Vicente Braga de Souza, Gislene Almeida Carvalho-Zilse

**Affiliations:** 1 Instituto Nacional de Pesquisas da Amazônia (INPA), Programa de Pós-Graduação em Genética, Conservação e Biologia Evolutiva (PPGGCBEv), Manaus, AM, Brazil.; 2 Instituto Nacional de Pesquisas da Amazônia (INPA), Coordenação de Sociedade, Ambiente e Saúde (COSAS), Laboratório de Micologia, Manaus, AM, Brazil.; 3 Universidade Estadual do Maranhão (UEMA), Departamento de Biologia, Laboratório de Genética e Biologia Molecular Warwick Estevam Kerr (LabWick), São Luís, MA, Brazil.; 4 Instituto Nacional de Pesquisas da Amazônia (INPA), Coordenação de Biodiversidade (COBIO), Grupo de Pesquisas em Abelhas (GPA), Manaus, AM, Brazil.

**Keywords:** Melipona interrupta, queens, caste segregation, larval food, Zygosaccharomyces

## Abstract

Stingless bees like *Melipona interrupta* play vital ecological roles and rely on diverse microbial communities in their larval food. This study investigated the impact of fungal diversity on larval development and caste differentiation. Fungi isolated from brood cell food were identified morphologically and molecularly, with *Fomitopsis* sp. and *Zygosaccharomyces* sp. showing high prevalence. Artificial larval rearing was conducted using sterilized and non-sterilized food inoculated with these fungi. *Zygosaccharomyces* sp. significantly enhanced survival rates and queen production, achieving results comparable to natural conditions, while *Fomitopsis* sp. had a modest effect. Statistical analyses confirmed significant associations between fungal treatments and larval outcomes. These findings underscore the functional role of fungi in *M. interrupta* larval nutrition and offer potential applications in sustainable meliponiculture.

## Introduction

Little is known about the microbial diversity associated with the development of *Melipona interrupta* and its impact on caste differentiation. The microbiota present in larval food plays a fundamental role in the ecological interactions of social bees, influencing critical biological processes such as survival and phenotypic differentiation. Studies suggest that fungi associated with larval food can interact with genetic-nutritional mechanisms, contributing to the formation of queens in stingless bee species such as *Melipona interrupta*.

Previous research has highlighted the role of fungi in the survival and larval development of social bees. For example, *Monascus ruber* significantly increased the larval survival rate of *Scaptotrigona depilis* under *in vitro* rearing conditions, suggesting its potential role in caste differentiation (Menezes et al., 2015; Paludo et al., 2018). Other fungi, such as *Zygosaccharomyces* sp., have also been associated with providing essential nutrients for bee development, reinforcing the hypothesis that these mutualistic associations directly contribute to colony nutrition and health (Meireles et al., 2022). Recent studies with *Melipona quadrifasciata*, *M. scutellaris*, *M. interrupta* and *M. seminigra* have identified fungi in larval food using molecular techniques, emphasizing their contribution to caste differentiation and larval development (Tiago et al., 2021; Santos et al., 2023). These findings underline the potential of fungal symbionts to modulate developmental processes in social bees.

In bees of the genus *Melipona*, caste differentiation occurs through a complex genetic-nutritional system. While all larval cells are of equivalent size and receive food of similar quality, the differentiation between queens and workers is influenced by environmental factors, such as nutrient availability (Hartfelder et al., 2006; Brito et al., 2013). Evidence suggests that the microbiota present in larval food may act as a critical factor modulating the gene expression involved in this process (Schumann et al., 2019). This interaction highlights the potential role of fungi as environmental modulators influencing the phenotypic fate of larvae (Menezes et al., 2007; Campos and Coelho, 1993). By understanding these mechanisms, it becomes evident how microbial diversity intersects with genetic determinants to shape caste outcomes in *Melipona* bees.

Understanding the interactions between fungal microbiota and the larval development of *Melipona interrupta* is essential for advancing sustainable colony management in meliponiculture. Beyond contributing to the conservation of native bees, this knowledge may enable innovative techniques to enhance productivity and manipulate castes. In a broader context, these discoveries open up opportunities for the bioprospecting of biotechnological compounds of industrial interest, given that microorganisms associated with bees can generate cost-effective and environmentally friendly substances.

In this study, we identified and characterized cultivable fungi associated with the larval food of *M. interrupta* using morphological and molecular techniques. Additionally, we tested the influence of these fungi on queen production under artificial conditions, providing new insights into microbial interactions and their application in colony management.

## Material and Methods

### Collection of biological material

The research was conducted with biological material obtained from three healthy colonies of *Melipona interrupta*, maintained in standardized boxes at the Scientific Meliponary of the Grupo de Pesquisas em Abelhas (GPA) located at the Instituto Nacional de Pesquisas da Amazônia (INPA), in Manaus, Amazonas, Brazil. The geographical coordinates for the site are 3°05’50.3”S latitude and 59°59’06.3”W longitude. This region is characterized by its tropical rainforest climate, providing a unique environment for the study of native bee species.

Three brood discs were collected aseptically using sterilized stainless-steel forceps (model XYZ-123, Fine Science Tools, Foster City, CA, USA). To avoid cross-contamination, separate forceps were used for each sample. After collection, the samples were immediately stored in sterilized bottles and transported to the laboratory within 1 hour.

Upon arrival at the laboratory, the samples were stored at 30 °C in a biological oxygen demand (BOD) incubator (model TE-391, Tecnal, Piracicaba, SP, Brazil) until further processing. For DNA extraction, the samples were homogenized in a TissueLyser II (Qiagen, Hilden, Germany) at 30 Hz for 2 minutes using sterile 5 mm stainless steel beads. The homogenate was then aliquoted for subsequent biochemical and microbiological analyses.

Quantitative and qualitative fungal analyses were conducted on brood cell samples collected from three healthy *Melipona interrupta* colonies maintained in standardized INPA boxes (GPA-INPA Meliponary, Manaus-AM, Brazil; 3°05’50.3”S 59°59’06.3”W). The colonies were monitored daily for 15 days, and samples were collected in triplicate at 1, 8, and 15 days post-laying to track fungal development during the larval feeding stages (Larva 1 and Larva 2). These time points were selected based on the developmental timeline of *Melipona* immatures (Amaral et al., 2010).

Brood cells were exposed to ultraviolet (UV) light for 15 minutes in a laminar flow cabinet (model Laminar Master 1300, Thermo Fisher Scientific, Waltham, MA, USA) before uncapping. Eggs or larvae were aseptically removed using sterilized stainless steel tweezers (VWR International, Radnor, PA, USA).

The food content, including fungal mass, was homogenized using a vortex mixer (Vortex Genie 2, Scientific Industries, Bohemia, NY, USA) and serial decimal dilutions (10⁻¹, 10⁻², 10⁻³) were prepared in sterile distilled water (Milli-Q^®^ IQ 7000, Merck Millipore, Burlington, MA, USA). Aliquots of 100 μL from each dilution were plated in triplicate on Potato Dextrose Agar (PDA; Kasvi, São José dos Pinhais, PR, Brazil) medium in sterile Petri dishes (90 mm; Fisher Scientific, Pittsburgh, PA, USA). Plates were incubated at 30 °C in a biological oxygen demand (BOD) incubator (model TE-391, Tecnal, Piracicaba, SP, Brazil) for 7 days and monitored daily for fungal growth. Fungal morphotypes were isolated and purified using the streak plate method under aseptic conditions.

### Morphological identification

Purified fungal isolates were subjected to microculture on slides following the method of Riddell (1950). Small fragments of fungal colonies were placed on PDA blocks on glass slides within sterile Petri dishes. The blocks were covered with sterile coverslips and incubated at 30 °C for 7 days in a humidified chamber. After incubation, fungal structures were stained with lactophenol cotton blue (Loba Chemie, Mumbai, India) and examined under an AxiosKop 40 light microscope (Carl Zeiss, Oberkochen, Germany).

Macromorphological characteristics, including colony color, shape, margin, and texture, were visually evaluated. Identification was conducted using morphological keys and descriptions provided by Barnett and Hunter (1972) and Seifert and Gams (2011).

### Molecular identification

Fungal DNA was extracted using a modified protocol by Ferrer et al. (2011). Briefly, fungal colonies were scraped from PDA plates and transferred to sterile microtubes containing 500 μL of lysis buffer (200 mM Tris-HCl, 250 mM NaCl, 25 mM EDTA, 0.5% SDS) and Proteinase K (20 mg/mL; Thermo Fisher Scientific, Waltham, MA, USA). Samples were incubated at 56 °C for 2 hours with gentle agitation.

Following lysis, 500 μL of phenol:chloroform:isoamyl alcohol (25:24:1; Sigma-Aldrich, St. Louis, MO, USA) was added, and the mixture was vortexed and centrifuged at 13,500 rpm for 10 minutes. The supernatant was transferred to new microtubes and DNA was precipitated with isopropanol at -20 °C for 30 minutes. After centrifugation, the pellet was washed twice with 70% ethanol, air-dried, and resuspended in 50 μL of elution buffer (Qiagen, Hilden, Germany). DNA integrity was assessed using a Nanodrop 2000 spectrophotometer (Thermo Fisher Scientific, Waltham, MA, USA).

The ITS (Internal Transcribed Spacer) region was amplified using primers ITS1 (5’-TCC GTA GGT GAA CCT GCG-3’) and ITS4 (5’-TCC TCC GCT TAT TGA TAT GC-3’) (White et al., 1990). PCR reactions were performed in a Veriti™ 96-Well Thermal Cycler (Applied Biosystems, Foster City, CA, USA) with a reaction mixture containing 1× PCR buffer (Thermo Fisher Scientific), 2.5 mM MgCl₂, 0.2 mM dNTP mix (Thermo Fisher Scientific), 0.5 μM of each primer, 1 U of Taq DNA polymerase (Thermo Fisher Scientific), and 2 μL of DNA template in a final volume of 25 μL. The cycling conditions included an initial denaturation at 94 °C for 5 min, followed by 40 cycles of denaturation at 94 °C for 30 s, annealing at 53 °C for 30 s, and extension at 72 °C for 1 min, with a final extension at 72 °C for 10 min.

PCR products were resolved on 1% agarose gels in 1× TBE buffer (Thermo Fisher Scientific) at 100V for 45 minutes. Gels were stained with SYBR^®^ Green (Invitrogen, Carlsbad, CA, USA) and visualized using a UVP GelDoc-It² Imaging System (Analytik Jena, Jena, Germany).

Sequencing was performed by ACTGene Análises Moleculares (Alvorada, RS, Brazil) using the Sanger method. Chromatograms were analyzed for quality control using Chromas software (Technelysium Pty Ltd., Brisbane, Australia). Sequences were aligned and compared to GenBank entries via BLAST (Basic Local Alignment Search Tool, NCBI).

### Experimental larval rearing

Sterilized Elisa-type microplates (Corning^®^, NY, USA) were used as artificial rearing cells. Each well received 156 μL of homogenized larval food collected from natural brood cells. The food was homogenized in a sterile Falcon^®^ tube (Thermo Fisher Scientific) and distributed aseptically using a pipette (Eppendorf Research^®^ plus, Hamburg, Germany).

Larvae (L1 stage) were transferred into wells using a fine-tipped sterilized brush (Pentel^®^, Tokyo, Japan). A volume of 1 μL (approximately 1 × 10⁶ CFU·mL⁻¹) of fungal suspension was applied. Artificial cells were placed in hermetically sealed plastic containers with a layer of sterile water to maintain 100% relative humidity (RH) for the first three days. Containers were incubated at 30 °C in a BOD incubator. RH was gradually reduced to 85% and then 75% using saturated solutions of KCl and NaCl, respectively, as described by Menezes et al. (2013).

Six experimental groups were established based on the treatment of larval food:

C1: Sterilized larval food treated with UV.
C2: Non-sterilized larval food.
T1: Sterilized larval food treated with UV + *Fomitopsis* sp.
T2: Non-sterilized larval food + *Fomitopsis* sp.
T3: Sterilized larval food treated with UV + *Zygosaccharomyces* sp.
T4: Non-sterilized larval food + *Zygosaccharomyces* sp.

Each treatment group consisted of 60 artificial cells with larvae. The development of larvae was monitored daily to assess caste segregation into workers and queens, as well as larval mortality.

## Statistical analyses

Morphotype frequencies were recorded as absolute and relative percentages to assess the distribution of fungal species among the collected samples. Statistical tests, including Chi-square tests (p < 0.05) and Fisher’s exact test (p < 0.05), were employed to evaluate associations between treatments and outcomes, ensuring the validity of the observed relationships. The mean and standard deviation were calculated for quantitative variables, and data normality was assessed using the Shapiro-Wilk test.

Statistical analyses were conducted using R version 4.2.1 (R Foundation for Statistical Computing, Vienna, Austria). Graphical representations, such as bar plots and scatter plots, were generated using the ggplot2 package (v3.3.5) to visually interpret the data trends and associations. For phylogenetic analysis, MEGA X (Molecular Evolutionary Genetics Analysis software, version 10.2.6, Pennsylvania State University, State College, PA, USA) was employed to construct maximum likelihood and neighbor-joining trees.

Phylogenetic trees were generated based on ITS and D1/D2 regions. Sequence alignment was performed using Clustal W integrated within MEGA X. The evolutionary model was selected based on the lowest Bayesian Information Criterion (BIC) score. Statistical support for clades was determined through 1,000 bootstrap replicates. Visualization of the phylogenetic tree was enhanced with iTOL (Interactive Tree of Life, v6.6.3, EMBL-EBI, Hinxton, UK).

The data were processed to ensure high reproducibility. Detailed descriptions of the masses, concentrations, and reagents used were recorded. For example, fungal DNA extraction utilized 500 μL of lysis buffer (200 mM Tris-HCl, 250 mM NaCl, 25 mM EDTA, 0.5% SDS) combined with Proteinase K (20 mg/mL; Thermo Fisher Scientific, Waltham, MA, USA). Each sample was prepared in triplicate to confirm consistency in results.

## Data accessibility

DNA sequences obtained in this study were submitted to GenBank under accession numbers PX456983 (FF2), and SUB15706952 (LV9), ensuring public accessibility for future research. 

To promote transparency and reproducibility, the study’s reagents and equipment were explicitly detailed. For instance, statistical analysis relied on R software (R Foundation, Vienna, Austria) and a Windows 10 workstation (model Inspiron 15, Dell Inc., Round Rock, TX, USA). All reagents, such as the lysis buffer and PCR primers, were sourced from reliable manufacturers. By providing comprehensive access to methodologies and raw data, this study ensures its findings can be independently verified and reproduced.

## Results

### Identification of fungal isolates from the food content of natural brood cells

**Figure 1-:**
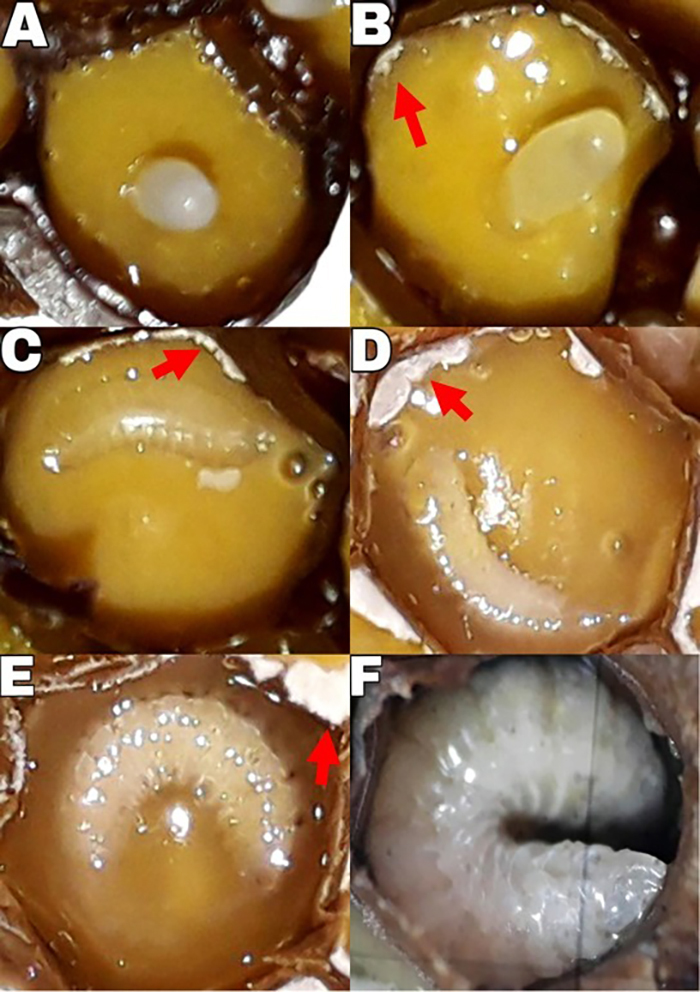
Fungal mass proliferation (indicated by red arrows), visually detected under a stereomicroscope (40X), throughout the brood development of *Melipona interrupta*. (A) Newly laid egg; (B) Egg about to hatch; (C) L1 larva; (D and E) L2 larva (beginning / end); (F) L3 larva.

During the early larval stages (L1), we observed distinct feeding behavior where the larvae consumed the fungal mass (Figure 1) by moving in circular patterns around themselves, positioning themselves at the center and consuming the fungal mass between the larva and the brood cell walls. All larvae completely consumed the visually detectable fungal mass in the brood cell (URL video available in internet resources section).

The fungal diversity observed in the larval diet of *M. interrupta* at different developmental stages was assessed by quantifying the colony-forming units (CFU) on PDA culture medium. The analysis aimed to determine the fungal community dynamics in the larval food, focusing on samples collected on days 1, 8, and 15 after queen oviposition. As shown in Table 1, a total of 10 fungal morphotypes, including both filamentous fungi (FF) and yeasts (LV), were identified. The CFU counts revealed that *Fomitopsis* (FF2) was the dominant taxon, with a significant increase in colony numbers over time, particularly at day 15. Other taxa such as *Zygosaccharomyces* (LV9) also showed substantial representation, with relatively stable CFU counts across the three time points. 

**Table 1 -:** Colony-Forming Units (CFU/mL) of Fungal Taxa (FF - Filamentous Fungi; LV - Yeast) Isolated on PDA Medium Inoculated with Natural Brood Cells of *Melipona interrupta*, at 1, 8, and 15 Days Post Queen Oviposition.

Morphotypes	CFU/mL (1 Day)	CFU/mL (8 Days)	CFU/mL (15 Days)	Total CFU
*Myceliasterilia FF1*	2	5	2	9
*Fomitopsis FF2*	19	35	41	95
*Myceliasterilia FF3*	0	2	2	4
*Oidiodendron FF4*	1	0	0	1
*Oidiodendron FF5*	0	0	1	1
*Cladosporium FF6*	2	2	1	5
*Myceliasterilia FF7*	5	2	2	9
*Oidiodendron FF8*	0	1	1	2
*Zygosaccharomyces LV9*	10	15	10	35
*Myceliasterilia FF10*	2	0	0	2

In contrast to *Fomitopsis* and *Zygosaccharomyces*, other fungal taxa exhibited lower and more variable CFU counts. For example, the Oidiodendron group, represented by FF4, FF5, and FF8, demonstrated minimal presence, with only sporadic occurrences across the samples. Interestingly, the “Mycelia sterilia” morphotypes, identified as FF1, FF3, FF7, and FF10, presented a consistent, albeit low, occurrence throughout the experiment. The total CFU across all taxa remained relatively stable between days 8 and 15, suggesting a microbial equilibrium is reached during the later stages of larval growth. 

The DNA extraction and amplification of the ITS1-5.8S-ITS2 region of rDNA and the D1/D2 region of rRNA were achieved for the two most frequent isolates (to *Fomitopsis* FF2 and *Zygosaccharomyces* LV9), resulting in bands of approximately 600 base pairs. The DNA sequences of the predominant fungal isolates were analyzed using Chromas to confirm sequence quality, and the BLASTN tool was employed to compare the sequences against the GenBank database, using a threshold of 97% similarity. 

**Figure 2-:**
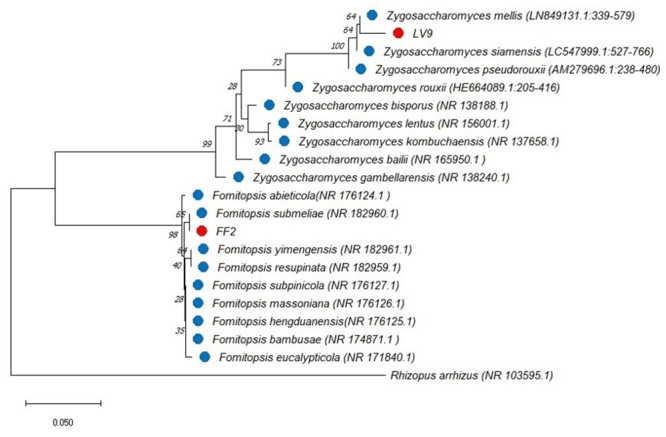
Tree generated using the neighbor-joining (NJ) method constructed for the internal transcribed spacer (ITS) molecular marker in the MEGA X software (Molecular Evolutionary Genetics Analysis). The selected fungal samples LV9 and FF2 (in red) were compared with sequences available in NCBI (in blue). *Rhizopus arrhizus* (NR.103595.1) was used as an out group.

A genetic distance tree was constructed using the neighbor-joining (NJ) method with MEGA X software. The bootstrap method (1000 replicates) was used to determine the percentage of trees in which the associated taxa clustered together, providing robust support for the phylogenetic relationships observed (Figure 2).

### Caste segregation from *M. interrupta* larvae treated with fungal inocula added to the food

In order to investigate the impact of fungal inoculation and food treatment with UV on the development of *M. interrupta* larvae, an experiment was designed comparing different treatments. The larvae were reared in artificial cells under laboratory conditions, and six groups were established: two control groups with sterilized (C1) and non-sterilized (C2) food, and four treatment groups, where food was either sterilized or non-sterilized, and inoculated with either *Fomitopsis* FF2 or *Zygosaccharomyces* LV9 (T1, T2, T3, T4). The results are presented in Table 2, which includes the number of surviving workers, queens, and males, in addition the dead individuals for each group. This setup aimed to test the hypothesis that fungal presence and treatment with UV would influence larval survival and caste differentiation.

**Table 2-:** Number of workers (W), queens (Q) and males (M) from *Melipona interrupta* larvae reared in artificial cells under six different larval food treatments. Each group was formed with 60 artificial cells with larvae.

Treatment	Number of Workers	Number of Queens	Number of Males	Total number of survivors (W+Q+M)	Total number of dead larvae
C1 - Laval food treated with UV	15	4	2	21	39
C2 - Larval Food	24	7	12	43	17
T1 - Larval food treated with UV + *Fomitopsis*	30	4	3	37	23
T2 - Larval food + *Fomitopsis*	25	7	11	43	17
T3 - Larval food treated with UV + *Zygosaccharomyces*	33	9	11	53	7
T4 - Larval food + *Zygosaccharomyces*	29	10	16	55	5

The treatment resulting in the highest number of dead larvae was **C1 - Larval food treated with UV**, with 41 dead larvae. The highest number of queens was observed in **T4 - Larval food + *Zygosaccharomyces*** , with 10 queens produced. The treatment yielding the most workers was **T3 - Larval food treated with UV + *Zygosaccharomyces*** , with 33 workers recorded.

To evaluate the association between treatments and outcomes (dead larvae, queens, male, and workers), a chi-square (χ²) test of independence and Fisher’s exact test were performed. The χ² test revealed a statistically significant association between treatments and the number of dead larvae (p = 0.002), indicating that the treatment method influenced larval mortality. For queen production, the p-value was 0.07, suggesting a trend but without statistical significance. Fisher’s exact test indicated that all experimental treatments significantly reduced mortality probability compared to the control group (C1). Treatment T1 presented an odds ratio (OR) of 0.338 (95% CI [0.15-0.75], p = 0.006), corresponding to a 66.2% reduction in mortality. T2 showed excellent efficacy (OR = 0.216, 95% CI [0.09-0.50], p = 0.0001), equivalent to a 78.4% decrease in mortality. The most pronounced effects were observed in treatments T3 (OR = 0.073, 95% CI [0.02-0.15], p = 1.799 × 10⁻⁹) and T4 (OR = 0.051, 95% CI [0.01-0.15], p = 6.852 × 10⁻¹¹), demonstrating 92.7% and 94.9% mortality risk reductions, respectively. These results highlight the potential influence of fungal inoculation and sterilization methods on larval outcomes. 

## Discussion

This study revealed groundbreaking biological insights into the role of fungi in the development and survival of *Melipona interrupta* under controlled conditions. Specifically, the inoculation of larval food with *Fomitopsis* sp. and *Zygosaccharomyces* sp. positively influenced survival rates and queen production, emphasizing a potential mutualistic relationship between these fungi and the bees. During the larval development phase, we identified nine filamentous fungi and one yeast in the larval food, with *Fomitopsis* sp. and *Zygosaccharomyces* sp. being predominant. These findings align with previous studies indicating that microbiota diversity supports social insect health (Mee and Barribeu, 2023). However, the novel association of *Fomitopsis* sp. with bees expands current knowledge on insect-microbiota interactions, suggesting new ecological and biotechnological roles. Furthermore, inoculation with *Zygosaccharomyces* sp. showed a superior impact on caste differentiation, potentially linked to ergosterol production, an essential precursor for ecdysteroid synthesis (Paludo et al., 2018). These results underline the ecological and functional significance of these fungi in bee biology and biodiversity conservation.

### Fungal isolation and diversity

The number of CFU associated with larval food increased significantly in later stages of larval development, confirming that a microbiota is crucial for the healthy growth of social insects. The identification of *Oidiodendron* sp. and *Cladosporium* sp. supports previous studies on their recurring presence in bee hives (Santos et al., 2019), while the novel detection of *Fomitopsis* sp. introduces a new dimension to our understanding of fungal diversity in social insects. These results are consistent with findings by Menezes et al. (2015), who reported that fungal symbionts contribute to food digestion and microbial regulation. Such associations reinforce the concept of a mutualistic relationship between bees and fungi, providing new perspectives on the role of microbiota in bee colonies.

### Fungi identification and associations

The inoculation experiments demonstrated that *Zygosaccharomyces* sp. enhanced larval development and caste differentiation more effectively than *Fomitopsis* sp., suggesting that ergosterol production plays a pivotal role in these processes. Similar findings were reported by de Paula et al. (2023), highlighting the ability of fungal symbionts to produce bioactive compounds that promote insect health. Interestingly, the presence of *Fomitopsis* sp., previously unreported in bee-related studies, opens opportunities for investigating its potential ecological functions and biotechnological applications. These results align with Motta and Moran (2024), who noted that specific microbiota compositions influence influence health, digestion, detoxification, immune system and beekeeping practices.

### Effects of fungi on survival and treatments

Our findings revealed that the inclusion of fungi in larval food significantly reduced mortality rates. *Zygosaccharomyces* sp. was particularly effective in enhancing survival, corroborating Paludo et al. (2018), who demonstrated that fungal symbionts provide essential nutrients and protect against pathogens. Furthermore, the ability of *Fomitopsis* sp. to contribute to survival suggests previously unexplored ecological roles. These insights expand our understanding of fungal symbioses and highlight the complex interactions between environmental conditions, microbiota composition, and host species. Comparing these results with Silva et al. (2023) underscores the importance of tailored microbiota interactions in optimizing colony health.

While this study provided significant insights, some limitations must be acknowledged. First, laboratory conditions may not fully replicate the natural ecological dynamics influencing bee-fungal interactions. Second, the reliance on cultivation techniques potentially excluded non-culturable fungi from the analysis, limiting the scope of microbiota identification. Future studies should integrate metagenomic approaches to capture the full diversity of microbial communities and validate these findings under field conditions. Investigating the biochemical pathways, particularly the role of ergosterols in caste differentiation, will further elucidate the mechanisms underlying these mutualistic relationships. Additionally, exploring the interactions between environmental variables and microbiota will provide a holistic understanding of these complex ecological systems.

## Conclusions

This study significantly advances the understanding of fungal roles in the biology of *Melipona interrupta*, highlighting their critical contributions to larval survival and caste differentiation. The identification of *Fomitopsis* sp. and *Zygosaccharomyces* sp. as key symbionts not only expands ecological knowledge but also opens avenues for biotechnological applications. For instance, the bioactive compounds produced by these fungi have potential applications in sustainable agriculture and pharmaceuticals. Furthermore, the findings emphasize the importance of fungi in conserving native bee populations, providing actionable insights for biodiversity management. By bridging gaps in microbial ecology and insect biology, this work lays the foundation for innovative strategies in conservation and sustainable development.

## Data Availability

DNA sequences obtained in this study were submitted to GenBank under accession numbers PX456983 (FF2), and SUB15706952 (LV9) ensuring public accessibility for future research.
